# Development and Validation of a Hypertension Risk Prediction Model Based on Particle Swarm Optimization–Support Vector Machine

**DOI:** 10.3390/bioengineering12030238

**Published:** 2025-02-26

**Authors:** Rou You, Qiaoli Tao, Siqi Wang, Lixing Cao, Kexue Zeng, Juncai Lin, Hao Chen

**Affiliations:** 1 School of Medical Information Engineering, Guangzhou University of Chinese Medicine, Guangzhou 510006, China; yourou233@stu.gzucm.edu.cn (R.Y.); 20211110386@stu.gzucm.edu.cn (Q.T.); wangsiqi@stu.gzucm.edu.cn (S.W.); linjuncai468@stu.gzucm.edu.cn (J.L.); 2 Guangdong Provincial Hospital of Chinese Medicine, Guangzhou 510120, China; lixingcao@126.com; 3 Guangdong Provincial Second Hospital of Traditional Chinese Medicine, Guangzhou 510009, China; zengkexue@163.com; 4 Guangdong Provincial Engineering Technology Research Institute of Traditional Chinese Medicine, Guangzhou 510009, China

**Keywords:** hypertension, machine learning, support vector machine, particle swarm optimization, classification model

## Abstract

Background: Hypertension is a prevalent health issue, especially among the elderly, and is linked to multiple complications. Early and accurate detection is crucial for effective management. Traditional detection methods may be limited in accuracy and efficiency, prompting the exploration of advanced computational techniques. Machine learning algorithms, combined with optimization methods, show potential in enhancing hypertension detection. Methods: In 2022, data from 1460 hypertensive and 1416 non-hypertensive individuals aged 65 and above were collected from the Lujingdong Outpatient Department of the Guangdong Second Traditional Chinese Medicine Hospital. Support Vector Machine (SVM) and Particle Swarm Optimization–Support Vector Machine (PSO-SVM) models were developed, validated using the holdout method, and evaluated based on sensitivity, specificity, positive predictive value (PPV), accuracy, G-mean, F1 score, Matthews correlation coefficient (MCC), and the area under the curve (AUC) of the receiver operating characteristic curve (ROC curve). Results: The PSO-SVM model outperformed the standard SVM, especially in sensitivity (93.9%), F1 score (0.838), and AUC-ROC (0.871). Conclusion: The PSO-SVM model is effective for complex classifications, particularly in hypertension detection, providing a basis for early diagnosis and treatment.

## 1. Introduction

Hypertension is a leading modifiable risk factor for cardiovascular events and mortality globally. Data from the World Health Organization indicate that it affects over one billion people worldwide [1], with a high prevalence among the elderly in China, where 54.6% of the elderly population suffer from it [2,3,4]. Characterized by elevated systemic arterial blood pressure, hypertension often lacks obvious early symptoms but can cause severe complications, significantly impacting patients’ quality of life [5,6,7]. Early diagnosis and prevention are crucial for reducing its harm [8]. Previous research has identified numerous factors contributing to hypertension, including demographic, disease-related characteristics, laboratory test results, and external factors [9,10,11,12]. However, most studies rely on traditional statistical analysis methods, focusing on single risk factors and lacking comprehensive risk assessment and prediction. Moreover, the absence of effective predictive models makes it difficult for clinicians to make accurate diagnoses and treatment decisions in complex cases.

Traditional hypertension testing relies heavily on intermittent blood pressure measurements in a clinical setting, such as with a sphygmomanometer, but human error in the measurements as well as inconsistencies in the timing of the measurements can lead to inaccurate results. Intermittent measurements also somewhat overlook the effects of factors such as daily activities, stress, and diet on blood pressure. A single measurement may not accurately represent a person’s true hypertensive state. At the same time, traditional methods often fail to consider potential interactions between influences such as genetics, lifestyle, and environmental factors.

In recent years, machine learning has been widely used in healthcare, showing great potential in predicting health outcomes. Supervised learning algorithms, in particular, are commonly applied to predict disease incidence and patient prognosis [13]. Examples of successful applications of machine learning-based algorithms in different medical scenarios abound. For instance, Sheela and Arun [14] successfully utilized a hybrid PSO-SVM algorithm for COVID-19 screening and quantification, which not only demonstrated the practical application of PSO-SVM in healthcare, but also showed the potential of such algorithms in disease-related prediction tasks. Moreover, Mirmozaffari et al. [15] proposed a model to optimize transfer times for ischemic stroke patients, highlighting the importance of model-based approaches in improving the medical process. These studies further emphasize the broad application of advanced computational techniques in healthcare, which is in line with the trend of our research on hypertension risk prediction using machine learning methods. Many studies have explored the early detection and treatment of hypertension using various machine learning methods. For example, Du et al. [16] used logistic regression to analyze factors in Inner Mongolia for hypertension prediction, while Ren et al. [17] did similar work in Northern China. However, traditional machine learning methods like decision trees, random forests, and logistic regression have limitations in parameter optimization, handling nonlinear relationships, and managing high-dimensional medical data [18,19,20]. Logistic regression assumes linearity and independence among variables [21], which restricts its ability to capture complex interactions. Random forests are prone to overfitting with correlated features [22], reducing predictive accuracy.

Most current studies have focused on the application of basic mechanisms, for example, in relatively homogeneous populations, and there is a lack of comprehensive studies integrating multiple data sources (e.g., genetic data, lifestyle data, and continuous blood pressure monitoring data). Our study aims to address these gaps through the use of diverse and large-scale datasets, enabling the development of more robust prediction models applicable to a wider range of individuals. At the same time, we found that many current hypertension prediction models combined with machine learning are based on the application of a single machine learning algorithm, which limits the generalization of these models. To overcome this limitation, this study integrates Particle Swarm Optimization (PSO) with Support Vector Machines (SVM). SVM, based on statistical learning theory, is robust and efficient in medical prediction but is affected by suboptimal parameter selection [23,24]. On the other hand, PSO, a swarm intelligence-based optimization algorithm, can globally search the parameter space to improve model performance [25,26]. The integration of PSO and SVM can enhance parameter selection and better handle nonlinear, high-dimensional data [27].

Previous studies have shown the effectiveness of combining PSO with other algorithms. For example, Binh Tran et al. [28] proposed the PSO-LSRG algorithm to improve feature selection performance. El-Shafiey et al. [29] developed a GAPSO–RF hybrid model for better heart disease prediction. DONG et al. [30] achieved high accuracy in pathology image classification using a PSO-based algorithm. Selvarani et al. [31] enhanced pathology image analysis accuracy with PSO-SVM, and Saifullah et al. [32] explored the application of PSO in medical image segmentation. SM Hejazi et al. [33] showed that optimizing models can lead to better medical outcomes. These studies further validate our idea of using PSO-SVM to enhance the performance of hypertension risk prediction models, highlighting the potential of this approach in the medical field.

Therefore, building on these advancements, this study combines PSO and SVM to develop a hypertension recognition model that incorporates biomedical indicators, lifestyle factors, and environmental variables. By using PSO-SVM algorithms, the model can effectively handle high-dimensional data, improve computational efficiency, and enhance classification accuracy. This study also aims to refine parameter selection and risk feature screening, systematically addressing the complexities of high-dimensional data and improving classification performance. Ultimately, we aim to establish a robust PSO-SVM-based hypertension identification model to provide early prediction and intervention tools, reduce healthcare costs, and improve health outcomes.

## 2. Materials and Methods

### 2.1. Study Population

This study employs a cross-sectional research design, amassing data from elderly individuals aged 65 and above who participated in a complimentary health examination at the Lujingdong Outpatient Clinic of Guangdong Second Traditional Chinese Medicine Hospital from January to December 2022. The inclusion criteria for the subjects were the presence of a definitive diagnosis of hypertension and a complete set of pertinent medical indicators. This research has been approved by the Ethics Committee of Guangdong Second Hospital of Traditional Chinese Medicine (NO.Y202407-004-01).

### 2.2. Study Design and Participants

This study has amassed data from a cohort of 3458 subjects, acquiring a spectrum of sociodemographic characteristics (such as gender and age), medical histories, lifestyle factors (including smoking and alcohol consumption status), physical examination findings (encompassing height, weight, waistline, BP, and pulse rate), and biochemical markers, such as hemoglobin (HGB), leucocytes (LEU), fasting plasma glucose (FBG), serum alanine transaminase (ALT), serum aspartate transaminase (AST), total bilirubin (Tbil), serum creatinine (Scr), blood urea (BU), total cholesterol (TCHO), triglyceride (TG), low-density lipoprotein (LDL-C), high-density lipoprotein (HDL-C), and uric acid (UA).

### 2.3. Inclusion and Exclusion Criteria

We excluded participants with incomplete medical records that would affect the accurate determination of their hypertension status or the measurement of relevant variables. Also, individuals with acute severe diseases that could confound the relationship between risk factors and hypertension were excluded from the analysis.

In this study, the criteria for the inclusion of patients are as follows:

Hypertensive individuals: (1) individuals aged 65 years or older, with a systolic blood pressure (SBP) of 140 mmHg or higher and/or a diastolic blood pressure (DBP) of 90 mmHg or higher while not on anti-hypertension medication [34] and (2) those who have been previously diagnosed with hypertension and are currently undergoing treatment with anti-hypertension medications.

Non-hypertensive individuals: Those aged 65 years or older with a systolic blood pressure (SBP) below 140 mmHg and a diastolic blood pressure (DBP) below 90 mmHg, and no history of hypertension diagnosis.

### 2.4. Data Preparation

The selection of variables is critical in model construction and prediction [35,36,37]. Including inappropriate variables in the model may lead to misleading results, while an excessive number of variables could introduce multicollinearity, reducing predictive efficiency. Therefore, controlling the number of variables is essential to ensure the model’s reliability and performance.

In this study, the dataset included multiple variables such as age, gender, lifestyle factors, and biochemical indicators, comprising a total of 37 feature variables. The specific definitions of these variables are provided in Table 1. To ensure data quality and enhance analytical reliability, the following data preprocessing steps were undertaken:

(1) Variable Definition and Standardization: The dataset includes continuous variables (e.g., age, BP, and FBG) and categorical variables (e.g., gender and smoking status). The definitions and coding methods for categorical variables are provided in Table 1. For example, gender was coded as 0 for females and 1 for males, while smoking status was categorized by age groups or daily smoking quantity and encoded accordingly.

(2) Handling Missing Values: For variables with a missing rate below 30%, imputation methods were applied based on the variable type. Median imputation: used for skewed continuous variables (e.g., UA and ALT). Mean imputation: applied to continuous variables with approximately normal distributions (e.g., BMI). Mode imputation: used for categorical variables (e.g., diabetes status). Variables with a missing rate exceeding 30% were excluded from the analysis.

(3) Outlier Treatment: Outliers were identified and removed using the interquartile range (IQR) method, where values below or above were considered outliers. This helped to minimize bias in data distribution.

(4) Encoding and Numerical Transformation: Categorical variables (e.g., gender, dietary balance, and exercise habits) were processed using One-Hot encoding. Variables with more than five categories (e.g., daily smoking quantity and exercise duration) were grouped logically and converted into numerical values. Variables with differing units (e.g., lipid profile indicators) were converted and standardized to ensure consistency.

(5) Normalization: All numerical variables were standardized using the ‘StandardScaler’ from the ‘scikit-learn’ library. The ‘StandardScaler’ calculates the mean and standard deviation of each feature, transforming the data into a distribution with a mean of 0 and a standard deviation of 1. This effectively mitigates the impact of scale differences among various features, ensuring that each feature plays a balanced role in subsequent analyses. In the actual operation, the ‘fit_transform’ method of the ‘StandardScaler’ is first applied to the training set data. This not only computes the mean and standard deviation of the training set but also accomplishes the standardization transformation of the training set. As for the test set data, they are standardized via the ‘transform’ method, using the mean and standard deviation calculated from the training set. This approach prevents the interference of test set data in the training process, guaranteeing the accuracy and fairness of model evaluation. Following these preprocessing steps, 2876 valid samples with 37 feature variables were retained for further analysis.

### 2.5. Statistical Methods

The data analysis was conducted using SPSS version 22.0, R version 4.3.0 and PyCharm version 2020.1 software. Quantitative data are presented as medians with interquartile ranges (the 25th percentile and the 75th percentile), while qualitative data are represented by counts and proportions. The t-test and chi-square (χ2) test were employed to analyze the associations between different groups. Logistic regression analysis was performed on characteristics that showed statistically significant differences in univariate analysis to identify independent protective and risk factors for hypertension. A *p*-value of < 0.05 was considered to indicate statistical significance.

Using the statistically significant characteristics from the univariate analysis as independent variables, with the presence or absence of hypertension as the dependent variable, 80% of the dataset was randomly selected for the training set and 20% for the validation set. Predictive models were established using both the traditional SVM algorithm and the optimized Particle Swarm Optimization–Support Vector Machine (PSO-SVM) algorithm. Subsequently, the performance of the models was comprehensively evaluated.

## 3. Algorithm Principle

### 3.1. SVM Algorithm

The Support Vector Machine (SVM) is an efficient supervised learning algorithm, originally proposed by Vapnik and his colleagues [24] in 1995, primarily for solving classification problems. The central concept of SVM is to identify an optimal hyperplane that maximizes the margin between classes, thereby enabling effective data classification. This hyperplane serves as the decision boundary, and the margin represents a buffer zone between correctly and incorrectly classified samples. SVM excels in dealing with high-dimensional data, nonlinear issues, and imbalanced datasets and offers robust generalization capabilities. It holds significant advantages, particularly in the assessment of chronic disease risks, such as hypertension.

### 3.2. PSO Algorithm

PSO [38] is an algorithm that emulates the collective behaviors of biological entities such as flocking birds or schooling fish to find the optimal solution to a problem. The core mechanism of PSO is based on the collaboration and information sharing among individuals, known as particles, within a swarm. Inspired by the natural phenomena where individuals communicate and cooperate to enhance the adaptability and survival chances of the group, PSO has demonstrated effectiveness in parameter optimization problems due to its relatively simple parameter setting and rapid convergence. In PSO, each particle represents a potential solution in the solution space. The particles update their positions and velocities dynamically by tracking their own best position (personal best, pbest) and the best position achieved by any particle in the swarm (global best, gbest). This process allows for an efficient search for the optimal solution within the solution space [39]. “Pbest” refers to the best position or solution a single particle has found during its search process, representing the most optimal result achieved by that particle so far. “Gbest,” on the other hand, is the best solution identified by the entire swarm of particles, which reflects the collective exploration of the group and represents the optimal solution discovered during the entire search process. These two concepts, pbest and gbest, play a crucial role in guiding the particles to converge towards the optimal solution in the PSO algorithm. It is important to emphasize that “pbest” and “gbest” are concepts of parameter optimization within the algorithm. They are unrelated to the health status of patients and are solely used to guide the search process in the Particle Swarm Optimization algorithm. For example, imagine a flock of birds searching for food in a large field. Each bird represents a particle in the PSO algorithm. The birds communicate with each other about the best food-finding locations they have discovered. The best place a single bird has found on its own is like the ‘pbest’ in PSO, while the best place found by any bird in the whole flock is the ‘gbest’. The birds adjust their flight directions (like particles adjusting their velocities and positions) based on this information to find the food more efficiently. This is the basic idea behind how PSO emulates biological behaviors for optimization.

### 3.3. PSO-SVM Algorithm

The integration of PSO with SVM for non-probabilistic binary linear classification is indeed a powerful combination that has demonstrated its efficacy across various domains. The PSO-SVM method is favored for its ease of implementation and broad applicability, and it has shown superior performance and accuracy in numerous applications, including the classification of various diseases [14,40,41,42]. By combining PSO with SVM, one can harness the global search capabilities of PSO to optimize the critical parameters of the SVM, such as the penalty coefficient (C) and the kernel parameter (γ). This synergy not only enhances the efficiency of the parameter search but also bolsters the predictive performance of the SVM model on complex datasets.

In the context of an SVM model augmented by PSO, the optimization strategy is meticulously crafted to not only avoid entrapment in local optima but also to swiftly converge upon the global optimum. This capability significantly enhances the precision of classification or regression analysis in practical applications. In this study, we applied the PSO-SVM approach to hypertension risk prediction tasks. Hypertension, as a complex chronic disease, involves multiple interwoven influencing factors such as age, biochemical markers (e.g., ALT, Scr, and UA), and lifestyle variables. The intricate interactions among these features pose challenges for traditional machine learning models to effectively capture and model. To address this issue, we leveraged PSO’s global search capability to optimize the key parameters of SVM (C and γ), enhancing the model’s adaptability to high-dimensional nonlinear data and achieving greater predictive accuracy and robustness. Here is a descriptive overview of the algorithm:

In an M-dimensional feature space, N particles are randomly generated, where each particle symbolizes a set of parameters for a potential SVM model. For each particle, specifically the i(i∈N)i particle, the performance of its coordinates $X_{i}\leftarrow \left(x_{i1},x_{i2},\cdots ,x_{im}\right)$ is evaluated under the objective function, and both its personal best solution $P_{i}\leftarrow \left(p_{i1},p_{i2},\cdots ,p_{im}\right)$ and the global best solution $P_{g}\leftarrow \left(p_{g1},p_{g2},\cdots ,p_{gm}\right)$ are documented.

According to the PSO algorithm, the velocity of a particle $V_{i}\leftarrow \left(v_{i1},v_{i2},\cdots ,v_{im}\right)$ is updated based on the following formula:

(1) $$v_{im}^{k+1}\leftarrow {\omega v}_{im}^{k}+c_{1}{rand}_{1}()\left(p_{im}^{k}-x_{im}^{k}\right)+c_{2}{rand}_{2}()\left(p_{gm}^{k}-x_{im}^{k}\right)$$

In Formula (1), each parameter has the following meaning:

$v_{im}^{k}$: The velocity of particle i in the m−th dimension at the k−th iteration, indicating the speed of the particle in this dimension at a specific iteration.

ω: The inertia weight, set as a constant in the PSO algorithm. It maintains particle movement continuity and determines the influence of the previous velocity in the current update.

$c_{1}$ and $c_{2}$: Cognitive and social learning factors. $c_{1}$ reflects the impact of an individual particle’s experience on its velocity adjustment, while $c_{2}$ represents the influence of the swarm’s experience on the particle’s movement.

${rand}_{1}()$ and ${rand}_{2}()$: Random functions generating numbers between 0 and 1, introducing randomness to help the algorithm explore the solution space and avoid local optima.

$p_{im}^{k}$: The personal best position of particle i in the m−th dimension at the k−th iteration, being the best-found position of the particle in this dimension so far.

$x_{im}^{k}$: The current position of particle i in the m−th dimension at the k−th iteration.

$p_{gm}^{k}$: The global best position in the m−h dimension at the k−th iteration, which is the best position discovered by the whole swarm up to this iteration.

Typically, the inertia weight ω is set within a range, such as [0.4, 0.9]. A larger ω value makes the particles move more like they did in the previous iteration, which is beneficial for global exploration, helping the algorithm search a wider area of the solution space. A smaller ω value emphasizes local exploitation, making the particles focus more on refining the solutions around the current best-known positions. The cognitive and social parameters $c_{1}$ and $c_{2}$ are usually set to around 2. They control how much the particle is influenced by its own past experience ($c_{1}$) and the collective experience of the swarm ($c_{2}$). The random numbers ${rand}_{1}$ and ${rand}_{2}$ introduce randomness, preventing the algorithm from getting stuck in local optima.

(2) $$v_{im}\leftarrow \left\{\begin{array}{c} v_{max}\;\;\;\;\;\;\;if\;\;v_{im}>v_{max}\\ -v_{max}\;\;\;\;\;\;if\;\;v_{im}<{-v}_{max} \end{array}\right.$$

Within the framework of the PSO algorithm, the inertia weight ω is set to a constant value to maintain the continuity of particle movement. The iteration counter k keeps track of the current round of the algorithm. The cognitive and social learning factors $c_{1}$ and $c_{2}$, respectively, reflect the influence of individual and social experience on the adjustment of particle velocity. According to Formula (2), when greater than 0, the maximum value of $v_{im}$ is $v_{max}$. If $v_{im}$ is greater than $v_{max}$, then $v_{im}$ equals $v_{max}$; if $v_{im}$ is less than ${-v}_{max}$, then $v_{im}$ equals $v_{max}$. The global best solution $P_{gm}$ represents the best solution found in the history of the swarm and guides the flight path of the particles. The personal best solution is the optimal state that each particle has achieved during the search process. As the global best solution is discovered, the personal best solutions of the particles are also adjusted accordingly. Similarly, the global position of the particles is updated based on the best solution of the swarm. These updating processes follow specific formulas for velocity and position adjustments:

(3) $$x_{im}^{k}\leftarrow x_{im}^{k}+v_{im}^{k}$$

The local and global optimal solutions obtained through the optimization process are applied to the kernel function of the SVM, and the penalty coefficient *C* is introduced into the objective function of the SVM to achieve precise model adjustment and margin maximization:

(4) $$f\left(w\right)\leftarrow \frac{{\left\|\underset{\omega }{\to }\right\|}^{2}}{2}+C{\left(\sum_{i=1}^{N}\xi_{i}\right)}^{K}$$

in which $\underset{\omega }{\to }$ represents the normal vector to the decision plane, C is the coefficient used to measure the degree of penalty for misclassification, and ξi denotes the allowable error margin or slack variable. Through the fine-tuning of the objective function, we are able to maximize the interval between data points, thereby achieving the most accurate classification effect when processing hypertension data.

### 3.4. Evaluation of Machine Learning Prediction Model

**Figure 1:**
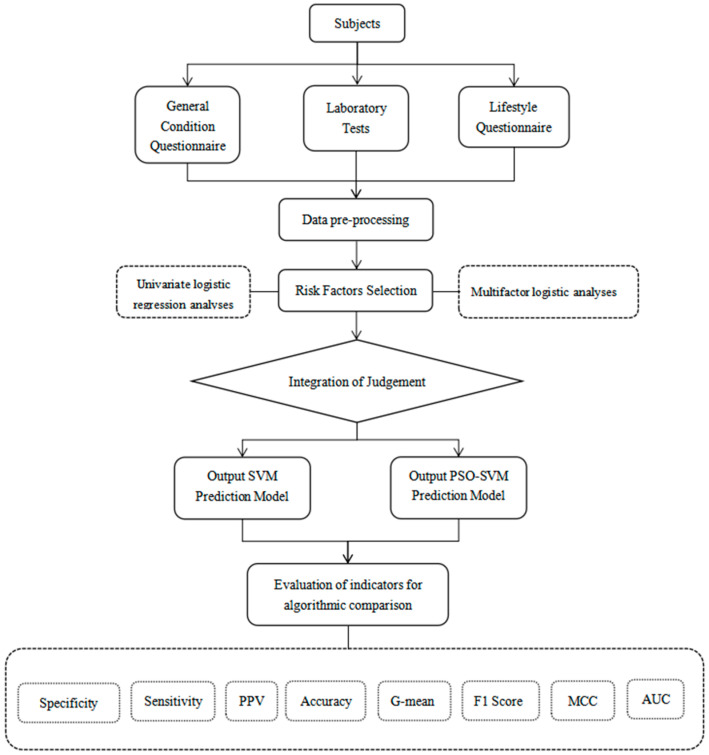
This study’s workflow diagram illustrates the process, including data collection, preprocessing, and risk factor selection, followed by the development of SVM and PSO-SVM models. Model performance is compared using evaluation metrics such as specificity, sensitivity, accuracy, and AUC. PPV, positive predictive value. MCC, Matthews correlation coefficient. AUC, area under the curve.

This study employed multiple evaluation criteria to measure the performance of the model, encompassing sensitivity, specificity, positive predictive value (PPV), accuracy, G-mean, F1 score, Matthews correlation coefficient (MCC), and the area under the curve (AUC) of the receiver operating characteristic curve (ROC curve). The research methodology is depicted in Figure 1.

## 4. Results

### 4.1. Screening of Influencing Factors

#### 4.1.1. Demographic Characteristics of the Study Population

This study included a total of 2876 cases based on the inclusion and exclusion criteria, with 1460 cases diagnosed with hypertension and 1416 cases without hypertension. The average ages of the hypertension and non-hypertension groups were (73.78 ± 7.08) years and (70.94 ± 5.56) years, respectively. In terms of age groups, 40.3% of the hypertensive patients were in the 65–70-year-old range, 26.9% were in the 71–75-year-old range, and 32.7% were 76 years or older. For non-hypertensive individuals, the corresponding proportions were 59.7%, 22.9%, and 17.3%, respectively. The proportion of females within the hypertension patient group was 59.3% (865 out of 1460), which was higher compared to the non-hypertension group where the female proportion was 56.9% (806 out of 1416).

#### 4.1.2. Differential Parameter Analysis Between Hypertension and Non-Hypertension Groups

Furthermore, hypertension patients demonstrated higher values in various parameters compared to the non-hypertension group, including age, pulse rate, weight, waistline, BMI, history of coronary heart disease, LEU, FBG, ALT, Scr, BU, TG, and UA, as presented in Table 2.

### 4.2. Univariate Analysis

Univariate analysis revealed that 29 factors, encompassing age, pulse, blood pressure, height, body weight, waist circumference, body mass index (BMI), lifestyle behaviors, medical history, and laboratory test results, were deemed to be influential in the development of hypertension (with *p* < 0.05), as presented in Table 2.

### 4.3. Logistic Regression Analysis

Logistic regression analysis was performed, with the presence of hypertension as the dependent variable (yes = 1, no = 0) and the indicators with statistically significant differences in the univariate analysis as the independent variables. The results identified age, LSBP, LEU, fasting blood sugar, serum alanine aminotransferase, serum creatinine, and uric acid as independent risk factors for hypertension, while gender (male) and total cholesterol were determined to be independent protective factors for hypertension, as presented in Table 3.

### 4.4. Modeling Analysis

**Figure 2:**
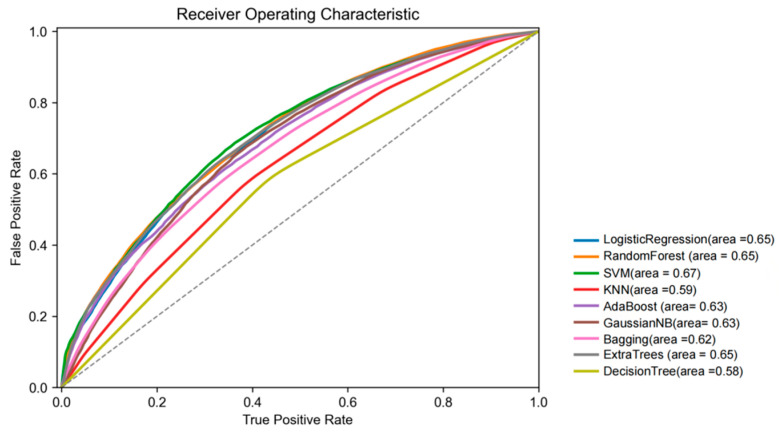
This ROC curve demonstrates that the SVM model outperforms the other 9 models (AUC = 0.67).

A random sampling strategy was applied to divide the dataset, with 80% allocated to the training set and 20% to the validation set. All statistically significant variables were included as predictors, with the presence of hypertension serving as the outcome variable. Nine machine learning methods were employed to construct hypertension classification models. The results (Figure 2) revealed that the SVM model achieved the highest AUC value (0.672), making it the selected baseline model. However, the initial SVM model demonstrated moderate classification performance, with a sensitivity of 0.596, a positive predictive value (PPV) of 0.637, an accuracy of 0.623, a geometric mean (G-mean) of 0.616, an F1 score of 0.616, a Matthews correlation coefficient (MCC) of 0.248, and an AUC value of 0.672 (Table 4).

To enhance the model’s performance, the PSO algorithm was applied to fine-tune the SVM model’s critical hyperparameters (C and  γ). During the PSO initialization stage, 50 particles were generated, with each particle representing a potential parameter combination. The search ranges for parameters C  and  γ were set to [$10^{-3}$, $10^{3}$] and [$10^{-3}$, $10^{1}$], respectively, to ensure coverage of the optimal solution space. Over a maximum of 50 iterations, each particle dynamically updated its velocity and position based on its historical best position and the global best position. The AUC value of the validation set was used as the fitness function to evaluate each particle’s performance.

The PSO algorithm’s convergence criteria were defined as either achieving less than a $10^{-4}$ change in the global best AUC value for five consecutive iterations or reaching the maximum iteration count. Convergence was achieved at the 10th iteration, with the global optimal parameters determined as C = 1.9436681 and γ = 0.10196975.

**Figure 3:**
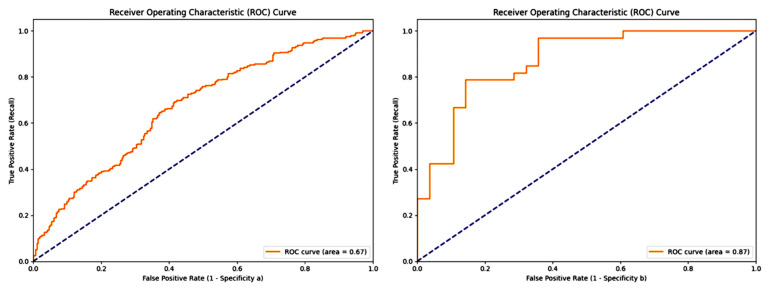
The ROC curves of the two models are presented. The SVM model. ROC curve illustrating the model’s ability to discriminate between classes. The gray dashed line indicates the random guessing baseline (AUC = 0.5). (**a**) achieves an AUC of 0.67, whereas the PSO-SVM model (**b**) exhibits superior performance with an AUC of 0.87.

When the SVM model was retrained using the optimized parameters, the PSO-SVM model exhibited significant performance improvements. The sensitivity increased to 0.939, positive predictive value (PPV) to 0.756, accuracy to 0.656, geometric mean (G-mean) to 0.715, F1 score to 0.838, Matthews correlation coefficient (MCC) to 0.618, and AUC value to 0.871. These results demonstrate that the PSO-optimized SVM model achieved superior classification performance and practical application potential in hypertension risk prediction tasks (Table 4, Figure 3).

To illustrate the improvements achieved through PSO optimization, Table 5 presents a comparison of the SVM model parameters before and after optimization.

We employed Least Absolute Shrinkage and Selection Operator (LASSO) regression to select and reduce the dimensionality of the 29 variables with statistical differences in Table 2. As the logarithm of (λ) increases, the average standard error rises, and the normalized coefficients of the 29 candidate variables are compressed to varying degrees until they all become zero [43]. The current results indicate that when the lambda for the minimum standard error is 0.01, the binomial model’s categorical variables consist of nine predictors. Ultimately, we identified the predictive variables for machine learning modeling to include age, male sex, LSBP, LEU, FBG, ALT, Scr, TCHO, and UA.

### 4.5. Visualization of the Prediction Model

**Figure 4:**
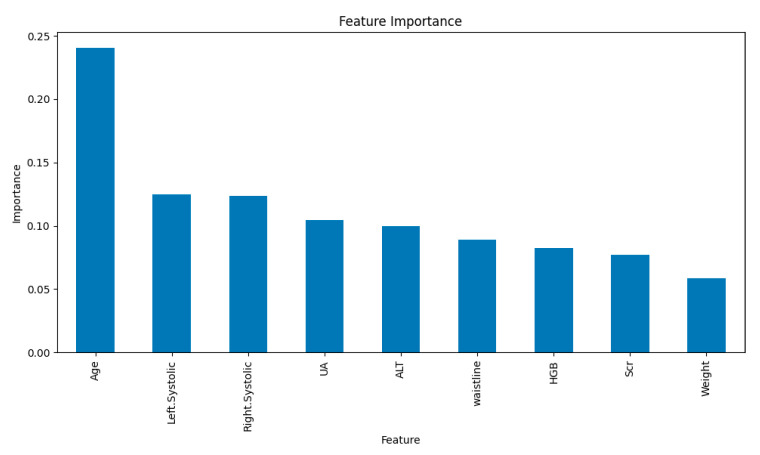
The bar chart displays the ranking of feature importance, where age has the highest importance, followed by left systolic blood pressure, right systolic blood pressure, UA, and ALT. Other features, including waistline, HGB, Scr, and weight, show relatively lower importance.

The results of the relative importance scoring, as displayed in Figure 4, reveal that among the nine statistically significant features included, age has the highest relative importance score, indicating that age is likely the most influential factor in the development of hypertension. Furthermore, LSBP, RSBP, UA, and ALT make substantial contributions to the model, and waistline, HGB, and Scr also contribute to a certain extent, whereas weight contributes minimally to the model.

**Figure 5:**
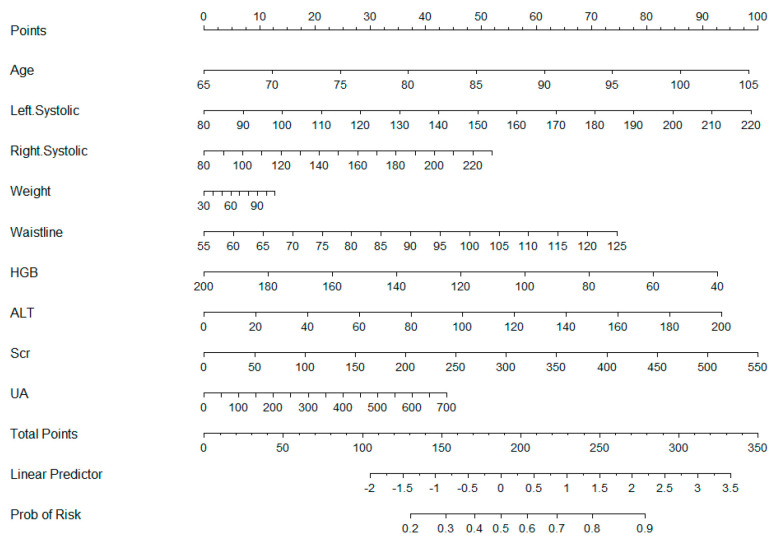
The nomogram illustrates the construction of a hypertension risk prediction model, where points are assigned to features such as age, left systolic blood pressure, right systolic blood pressure, weight, waistline, HGB, ALT, Scr, and UA. The total points are used to predict the linear predictor value and the corresponding probability of hypertension risk.

As shown in Figure 5, we have developed a nomogram to predict the risk of developing hypertension using nine predictive indicators: age, LSBP, RSBP, weight, waistline, HGB, ALT, Scr, and UA. The longer the line, the greater the risk factor for developing hypertension. Within the nomogram, each predictive factor has an associated “point” value. By summing the points for the nine predictive factors, a total score is obtained. Based on this total score, a corresponding percentage risk value can be derived, thereby ascertaining the risk of developing hypertension.

## 5. Discussion

This study developed and evaluated two machine learning models for hypertension risk prediction: SVM and PSO-SVM. The results demonstrated that PSO-SVM significantly outperformed the traditional SVM model across several critical metrics, including an AUC of 0.871, an F1 score of 0.838, an accuracy of 0.803, and a sensitivity of 0.939. The marked improvement in performance highlights the effectiveness of the PSO algorithm in optimizing hyperparameters, thereby significantly enhancing the classification capability of the SVM model.

PSO optimizes the SVM parameters through a unique mechanism. Each particle in PSO represents a potential set of SVM parameters. Initially, the particles are randomly distributed in the parameter space. As the algorithm iterates, the particles update their velocities and positions based on their own pbest and the gbest. For example, in our study, during the first few iterations, the particles exploring the values of the penalty coefficient and kernel parameter gradually moved towards more optimal regions. When the particles approached the optimal values, the performance of the SVM model, as measured by the AUC, started to improve significantly. The process of parameter optimization was a dynamic one, with the particles continuously adjusting their search directions until a satisfactory solution was reached.

We employed LASSO regression for feature selection, which, compared to ordinary least squares regression, more effectively manages multicollinearity and overfitting among variables. It is recognized as a comprehensive approach that aids in identifying effective predictors of hypertension risk [44]. Through LASSO regression analysis, nine critical predictors were selected from 29 candidate variables: age, LSBP, RSBP, weight, waistline, HGB, ALT, Scr, and UA. Among these, age emerged as the most significant risk factor, aligning with previous findings that advancing age is an independent major risk factor for hypertension [45]. However, results from other studies have varied. For instance, Rust et al. [46] identified high salt intake as a primary risk factor for hypertension, while Xu YZ et al. [47] found that BMI and waist circumference were independent risk factors for hypertension in female and male Kazakh populations, respectively, and also highlighted the role of abnormal total cholesterol levels in vascular diseases, including hypertension. Notably, in our study, waist circumference and body weight contributed less significantly, and smoking and alcohol consumption showed minimal importance in the model—particularly smoking, despite its established causal link to hypertension [48]. This discrepancy may stem from differences in study populations. Our study focused on individuals aged 65 and above residing in community settings, where activities are largely centered on communal health initiatives, such as educational lectures, group exercises, and health awareness campaigns. With well-implemented management and health education programs in local communities, certain risk behaviors, such as high salt intake, smoking, or poor dietary habits, may have reduced prevalence, which could partially explain the lower importance of these features. In the feature importance evaluation (Figure 3), left and right systolic blood pressure, UA, and ALT emerged as major contributors. UA, in particular, has been recognized in previous studies as closely linked to the risk of hypertension and has been incorporated into multiple risk prediction models with notable improvements in predictive performance [49,50,51]. Similarly, systolic blood pressure (whether left or right) is a critical variable in numerous risk models, consistently identified as essential for evaluating the impact of blood pressure changes on hypertension risk. ALT, a liver function marker, has also frequently appeared in hypertension prediction models, particularly those associated with metabolic syndrome, reflecting the intricate link between metabolic abnormalities and hypertension. Earlier research predominantly focused on basic physiological parameters, such as body weight and family history, with limited consideration of liver function markers like ALT or UA. The inclusion of these features provides a more comprehensive evaluation of hypertension risk, especially in complex clinical settings involving multiple comorbidities, where their consideration may enhance predictive accuracy [52]. Overall, the risk factors for hypertension remain a subject of debate. Therefore, further longitudinal analyses are required to deepen our understanding and confirm these findings.

Our hypertension dataset has its own characteristics. It is a high-dimensional dataset with 37 initial features, which can pose challenges such as the “curse of dimensionality”. PSO-SVM, through the combination of PSO-based parameter optimization and LASSO-based feature selection, effectively reduces the negative impact of high dimensionality. For example, LASSO regression helped to select the most relevant features, reducing the number of dimensions that the SVM had to handle. Meanwhile, PSO optimized the SVM parameters to better adapt to the remaining features. In addition, the data may contain noise due to measurement errors or individual variations. PSO-SVM, with its optimized parameters, is more robust to this noise, as it can better generalize from the training data to new, unseen data.

In the realm of modeling, machine learning, a subfield of artificial intelligence, focuses on training systems to learn from data and accurately predict future outcomes [53]. Beyond the global search capabilities of the PSO-SVM algorithm, other optimization techniques have also demonstrated significant potential in medical data processing. For instance, Wang et al. [54] proposed a Whale Optimization Algorithm (CMWOA) based on a chaotic multi-group strategy, achieving notable performance improvements in breast cancer and diabetes diagnosis using SVM. Shimpi et al. [55] enhanced the accuracy of diabetes prediction through an optimized hybrid classifier. Song et al. [56] improved SVM performance in handling complex datasets using a modified PSO algorithm, while Gao et al. [57] integrated Grey Wolf Optimization and PSO to address missing fasting glucose data, thereby enhancing the robustness of predictive models. When comparing these algorithms with PSO-SVM in the context of hypertension risk prediction, CMWOA, for example, has a different way of exploring the parameter space. It mimics the hunting behavior of whales, which might be more suitable for problems with different data distributions. In our hypertension data, which have a specific set of relationships between features and a particular level of noise, PSO-SVM showed better performance in terms of sensitivity and F1 score. However, CMWOA might be more efficient in terms of computational complexity for some datasets. For high-volume datasets, CMWOA’s multi-group strategy could potentially explore the parameter space more quickly. On the other hand, PSO-SVM is more straightforward to implement and has a better balance between exploration and exploitation in our data scenario. These studies collectively underscore the potential of optimization algorithms in improving model accuracy, managing high-dimensional nonlinear data, and providing critical support for clinical diagnosis and decision making.

This study employed PSO for optimization based on similar considerations. Traditional SVM models often suffer from performance limitations when parameters, such as the penalty coefficient (C) and kernel function parameter (γ), are improperly selected, particularly when dealing with high-dimensional and nonlinear data. PSO, through its global search capabilities and dynamic adjustment of individual optimal solutions, effectively explores the parameter space to identify superior solutions, thereby enhancing classification performance and generalization ability. In this study, the initial SVM model achieved an AUC of 0.672, a Se of 0.596, and an F1 score of 0.616, reflecting suboptimal performance. Following PSO optimization, the AUC significantly improved to 0.871, the sensitivity increased to 0.939, and the F1 score rose to 0.838. These results demonstrate the effectiveness of PSO in parameter optimization, significantly strengthening SVM’s adaptability to high-dimensional nonlinear data. Additionally, the PSO-optimized model more precisely captured interactions among features, effectively identifying and modeling the contributions of core variables such as age, LSBP, and UA. Compared to the traditional SVM, the PSO-SVM model exhibited substantial improvements in both training efficiency and predictive accuracy. These findings further confirm the potential of optimization algorithms in handling complex medical data, offering a more precise and feasible solution for hypertension risk prediction.

Furthermore, this study possesses several notable strengths. Firstly, it demonstrates the feasibility of employing optimization algorithms to enhance traditional machine learning models for hypertension risk prediction. Secondly, we conducted a comprehensive analysis of the economic feasibility and non-invasiveness of the selected variables, facilitating the practical application of the model. The candidate factors for model construction encompass a wide range, including sociodemographic characteristics, lifestyle factors, and laboratory test parameters.

## 6. Limitations

The limitations of this study warrant careful consideration. Firstly, this research was conducted using a single-center dataset comprising community-dwelling elderly individuals, with a limited sample size and relatively homogeneous age range (65 years and older). While this sample holds specific representativeness for the target population, it may restrict the generalizability of the findings to broader populations or younger age groups. Secondly, the analysis relied on self-reported data for certain variables, which could introduce recall bias or inaccuracies. Much of the data were collected through questionnaires, and elderly participants’ understanding of lifestyle factors and long-term recollections might be prone to inaccuracies (e.g., dietary habits such as the balance of meat and vegetables), potentially introducing bias. Finally, although the PSO-SVM model demonstrates significant advantages over traditional SVM, this study did not explore alternative optimization algorithms or hybrid methods that could further enhance predictive performance. As new methodologies and optimization techniques continue to evolve, future research should focus on exploring their application and effectiveness in improving hypertension prediction models.

## 7. Conclusions

The PSO-SVM model, based on key feature variables such as age, male sex, LSBP, LEU, FBG, ALT, Scr, TCHO, and UA, demonstrated exceptional performance in predicting hypertension risk. This model not only enables early identification of hypertension but also provides actionable feedback to optimize intervention strategies, thereby improving patient outcomes and reducing disease burden. Our study highlights the critical role of key risk factors in hypertension prediction, offering a scientific basis for precise identification of high-risk populations and efficient resource allocation. Furthermore, this research underscores the potential of PSO-SVM in handling high-dimensional, nonlinear medical data, showcasing its broad applicability in medical diagnosis and predictive tasks.

## Figures and Tables

**Table 1:** Definitions and descriptions of variables included in the dataset.

Variable	Definition	Variable	Definition
Age	Age	Hight	Hight
Temperature	Temperature	Weight	Weight
Respiratory Rate	Respiratory rate	Waistline	Waistline
Pulse Rate	Pulse rate	Age of smoking initiation	0: no; 1: <18; 2: 19–29; 3: >30
Gender	0: female; 1: male	HGB	Hemoglobin, (g/mol)
BMI	Body mass index = weight (kg)/height (m)^2^	LEU	Leucocyte (white blood cell), (10^9^/L)
Well-Balanced Diet	0: no; 1: yes	FBG	Fasting plasma glucose, (mmol/L)
LSBP/RSBP	Left systolic blood pressure/right systolic blood pressure, mmHg	ALT	Serum alanine transaminase, (U/L)
LDBP/RDBP	Left diastolic blood pressure/right diastolic blood pressure, mmHg	AST	Serum aspartate transaminase, (U/L)
Exercise	0: no; 1: yes	Tbil	Total bilirubin, (μmol/L)
Smoking	0: no; 1: yes	Scr	Serum creatinine, (μmol/L)
Drinking	0: no; 1: yes	BU	Blood urea, (mmol/L)
Diabetes Mellitus	0: no; 1: yes	TCHO	Total cholesterol, (mmol/L)
Hyperlipidemia	0: no; 1: yes	TG	Triglyceride, (mmol/L)
Fatty Liver Disease	0: no; 1: yes	LDL-C	Low-density lipoprotein, (mmol/L)
Coronary Artery Disease	0: no; 1: yes	HDL-C	High-density lipoprotein, (mmol/L)
Duration of Each Exercise	0: no; 1: <30 min; 2: <60 min; 3: >60 min	UA	Uric acid, (μmol/L)
Number of Cigarettes Smoked per Day	0: no; 1: <10; 2: 10–20; 3: 21–30; 4: >31		

Note: In this study, the “exercise” variable is defined as a binary value where 1 indicates that the elderly subject (aged 65 and above) engaged in regular physical activity, mainly consisting of simple aerobic exercises such as walking and tai chi. Given the relatively homogeneous nature of exercise types and intensities among this age group, with most activities being of a gentle nature due to their physical conditions, a more detailed intensity-based classification was not deemed necessary. This categorization was made to capture the overall participation in physical activity, which is known to have a significant impact on health outcomes in this population.

**Table 2:** Univariate analysis of the baseline data of the hypertension and non-hypertension groups.

Index	Hypertension (n = 1460)	Non-Hypertension (n = 1416)	*p*-Value
General Information (M,QL,QU)			
Age	73.79 (68.00, 78.00)	70.9 (67.00, 74.00)	<0.001
Temperature	36.46 (36.40, 36.50)	36.44 (36.40, 36.60)	0.467
Male (n, %)	595 (40.7)	610 (43.1)	0.201
Respiratory Rate	19.36 (18.00, 20.00)	19.3 (19.00, 20.00)	0.122
Pulse Rate	71.81 (64.00, 78.00)	70.09 (63.00, 76.00)	<0.001
LSBP	135.95 (125.00, 144.50)	129.41 (120.00, 137.00)	<0.001
LDBP	76.8 (71.00, 81.00)	75.31 (70.00, 79.00)	<0.001
RSBP	137.12 (126.00, 146.00)	130.55 (120.00, 140.00)	<0.001
RDBP	77.08 (71.00, 82.00)	75.46 (63.00, 81.00)	<0.001
Hight	157.55 (151.50, 163.50)	158.60 (153.00, 164.50)	0.001
Weight	60.92 (53.80, 67.80)	59.20 (52.20, 65.40)	<0.001
Waistline	85.03 (79.00, 90.00)	82.31 (76.50, 88.00)	<0.001
BMI	24.48 (22.50, 26.30)	23.47 (21.50, 25.40)	<0.001
Lifestyle (n, %)			
Well-Balanced Diet	1394 (95.4)	1369 (96.6)	0.097
Exercise	1127 (77.1)	1156 (81.6)	0.003
Smoking	110 (7.5)	161 (11.3)	0.002
Drinking	115 (7.8)	133 (9.3)	0.148
Duration of Each Exercise (n, %)			<0.001
<30 min	199 (13.6)	146 (10.3)	
<60 min	877 (60.0)	961 (67.8)	
>60 min	51 (3.4)	49 (3.4)	
Number of Cigarettes Smoked per Day (n, %)			0.013
<10	69 (4.7)	95 (6.7)	
10–20	68 (4.6)	86 (6.0)	
21–30	3 (0.2)	9 (0.6)	
>31	11 (0.7)	7 (0.4)	
Age of Smoking Initiation (n, %)			0.009
<18	57 (3.9)	62 (4.3)	
19–29	70 (4.7)	110 (7.7)	
>30	24 (1.6)	25 (1.7)	
Medical History (n, %)			
Diabetes Mellitus	290 (19.8)	319 (22.5)	0.028
Hyperlipidemia	47 (3.2)	43 (3.0)	0.115
Fatty Liver Disease	708 (48.4)	685 (48.3)	0.119
Coronary Artery Disease	249 (16.6)	203 (14.3)	0.002
Laboratory Tests (M,QL,QU)			
HGB (g/mol)	137.33 (129.00, 147.00)	139.1 (131.00, 147.00)	0.001
LEU (10^9^/L)	6.67 (5.49, 7.56)	6.24 (5.17, 7.09)	<0.001
FBG (mmol/L)	6.03 (5.07, 6.46)	5.78 (4.92, 5.91)	<0.001
ALT (U/L)	23.20 (15.30, 26.70)	21.67 (15.00, 24.50)	0.003
AST (U/L)	23.89 (18.90, 26.00)	23.93 (19.50, 26.10)	0.905
Tbil (μmol/L)	16.15 (12.95, 18.65)	16.85 (13.40, 19.30)	0.002
Scr (μmol/L)	74.62 (58.60, 83.50)	68.54 (55.60, 78.10)	<0.001
BU (mmol/L)	5.79 (4.64, 6.64)	5.49 (4.54, 6.24)	<0.001
TCHO (mmol/L)	5.25 (4.37, 6.06)	5.60 (4.84, 6.33)	<0.001
TG (mmol/L)	1.55 (0.94, 1.87)	1.42 (0.88, 1.69)	<0.001
LDL-C (mmol/L)	2.90 (2.23, 3.50)	3.15 (2.58, 3.71)	<0.001
HDL-C (mmol/L)	1.72 (1.42, 1.95)	1.81 (1.53, 2.06)	<0.001
UA (μmol/L)	346.12 (282.50, 400.85)	321.07 (264.00, 368.70)	<0.001

HGB, hemoglobin. BMI, body mass index. LEU, leucocyte. FBG, fasting plasma glucose. LSBP/RSBP, left systolic blood pressure/right systolic blood pressure. LDBP/RDBP, left diastolic blood pressure/right diastolic blood pressure. ALT, serum alanine transaminase. AST, serum aspartate transaminase. Tbil, total bilirubin. Scr, serum creatinine. BU, blood urea. TCHO, total cholesterol. TG, triglyceride. LDL-C, low-density lipoprotein. HDL-C, high-density lipoprotein. UA, uric acid.

**Table 3:** Logistic regression analysis.

Index	b	SE	OR (95%CI)	*p*-Value
Age	0.053	0.008	1.054 (1.038,1.07)	<0.001
Male	−0.677	0.139	0.508 (0.387,0.668)	<0.001
LSBP	0.019	0.005	1.020 (1.011,1.029)	<0.001
LEU	0.079	0.027	1.83 (1.026,1.142)	0.004
FBG	0.053	0.025	1.054 (1.004,1.107)	0.035
ALT	0.007	0.003	1.007 (1.001,1.014)	0.024
Scr	0.011	0.003	1.011 (1.005,1.017)	<0.001
TCHO	−0.238	0.079	0.788 (0.676,0.920)	0.003
UA	0.002	0.001	1.002 (1.001,1.003)	0.004

LSBP, left systolic blood pressure. LEU, leucocyte. FBG, fasting plasma glucose. ALT, serum alanine transaminase. Scr, serum creatinine. TCHO, total cholesterol. UA, uric acid.

**Table 4:** Predictive performance of each model for hypertension.

Model	Sensitivity	Specificity	PPV	Accuracy	G-Mean	F1 Score	MCC	AUC
SVM	0.596	0.651	0.637	0.623	0.616	0.616	0.248	0.672
PSO-SVM	0.939	0.643	0.756	0.803	0.843	0.838	0.618	0.871

SVM, Support Vector Machine. PSO-SVM, Particle Swarm Optimization–Support Vector Machine. PPV, positive predictive value. MCC, Matthews correlation coefficient. AUC, area under the curve.

**Table 5:** Performance comparison before and after parameter optimization.

Parameter	Initial Value	Optimized Value	Notes
C	1	1.9436681	Optimized by PSO
γ	0.1	0.10196975	Optimized by PSO
Kernel Function	rbf	linear	Optimized kernel choice
Tolerance (tol)	0.001	0.001	Default value
Max Iterations	−1	−1	Indicates no iteration limit
Random State	42	42	Ensures reproducibility of results

## Data Availability

The data presented in this study are available on request from the corresponding author due to privacy and ethical restrictions.

## Abbreviations

The following abbreviations are used in this manuscript:

SVM	Support Vector Machine
PSO	Particle Swarm Optimization
PSO-SVM	Particle Swarm Optimization–Support Vector Machine
PPV	Positive predictive value
MCC	Matthews correlation coefficient
ROC curve	Receiver operating characteristic curve
AUC	Area under the curve
BMI	Body mass index
HGB	Hemoglobin
LEU	Leucocyte (white blood cell)
FBG	Fasting plasma glucose
*LSBP/RSBP*	Left systolic blood pressure/right systolic blood pressure
*LDBP/RDBP*	Left diastolic blood pressure/right diastolic blood pressure
ALT	Serum alanine transaminase
AST	Serum aspartate transaminase
Tbil	Total bilirubin
Scr	Serum creatinine
BU	Blood urea
TCHO	Total cholesterol
TG	Triglyceride
LDL-C	Low-density lipoprotein
HDL-C	High-density lipoprotein
UA	Uric acid
LASSO	Least Absolute Shrinkage and Selection Operator
CMWOA	Chaotic Multi-Swarm Whale Optimizer Algorithm
CMWOA-SVM	Chaotic Multi-Swarm Whale Optimizer Algorithm–Support Vector Machine
